# Factors Influencing Milk Quality and Subclinical Mastitis in Dairy Herds Housed in Compost-Bedded Pack Barn System

**DOI:** 10.3390/ani13233638

**Published:** 2023-11-24

**Authors:** Karise Fernanda Nogara, Marcos Busanello, Queila Gouveia Tavares, Juliana Aparecida De Assis, Gustavo Freu, Marcos Veiga Dos Santos, Frederico Márcio Corrêa Vieira, Maity Zopollatto

**Affiliations:** 1 Department of Animal Science, Federal University of Paraná, Curitiba 80035-050, Brazil; queila.tavares@ufpr.br (Q.G.T.); julianassis05@gmail.com (J.A.D.A.); maity@ufpr.br (M.Z.); 2 Department of Agricultural Sciences, High Uruguay and Missions Regional Integrated University, Frederico Westphalen 98400-000, Brazil; marcosbusanello@hotmail.com; 3 Department of Animal Nutrition and Production, School of Veterinary Medicine and Animal Science, University of São Paulo, Pirassununga 13635-900, Brazil; gustavofreu@usp.br (G.F.); mveiga@usp.br (M.V.D.S.); 4 Biometeorology Study Group, Federal University of Technology—Paraná (UTFPR), Dois Vizinhos 85660-000, Brazil; fredericovieira@utfpr.edu.br

**Keywords:** housing system, microclimate conditions, bedding, dairy cows

## Abstract

**Simple Summary:**

Our study evaluated relationships among bedding characteristics, milk quality and composition, and subclinical mastitis occurrence in dairy herds housed in compost barn systems. Bedding moisture, temperature at 30 cm depth, and pH affected 63% of the variation in milk composition and quality. Environmental variables affected 77% of the variation in bedding variables, especially pH and the surface temperature. An increase of 1 °C for temperature at 30 cm depth resulted in a 0.6% reduction in the prevalence of subclinical mastitis. The bedding surface temperature at 22.3 °C resulted in the highest incidence of subclinical mastitis (~18.1%). Thus, controlling microclimatic conditions in compost barns optimizes the bedding composting process and ensures milk quality.

**Abstract:**

The compost-bedded pack barn (CBPB) system has been increasingly adopted by dairy farms due to its ability to enhance animal comfort and milk production. This study evaluated the associations among bedding characteristics, milk quality and composition, and subclinical mastitis (SCM) occurrence in dairy herds housed in CBPB systems. Over a period of six months, data related to milk quality and udder health and bedding sampling were collected from eight dairy farms. Monthly measurements of the bedding temperature and wind speed inside the CBPB were taken, while temperature and relative humidity data inside the CBPB were recorded using a datalogger. Bedding samples were subjected to analysis of moisture, pH, microbiological count, and carbon/nitrogen ratio. Data on milk composition (fat, protein, milk urea nitrogen, and total solids) and quality (somatic cell count and standard plate count) of bulk tank milk were obtained from DHIA results. Canonical correlation analyses were used to evaluate the association between the analyzed group variables, and linear regression models were used to identify associations between bedding characteristics and SCM occurrence in the studied herds. The bedding characteristics that most influenced milk composition and quality were moisture, temperature at 30 cm depth (T30), and bedding pH. Environmental variables played an important role in bedding composting, as they were closely related to the surface temperature and pH. Overall, 62.71% of the variation in milk quality and composition could be explained by the bedding variables, and 77.50% of the variation in the bedding variables was associated with environmental variables. Median SCM prevalence and incidence were 28.6 and 13.8%, respectively. An increase of 1 °C for T30 resulted in a 0.6% reduction in the prevalence of SCM. Additionally, the bedding surface temperature at 22.3 °C resulted in the highest incidence of SCM (~18.1%). Our results demonstrate the importance of controlling microclimatic conditions in the CBPB to optimize the bedding composting process and milk quality.

## 1. Introduction

The use of confined systems for housing of dairy cows has been growing among dairy farmers, with the aim of optimizing land use, ensuring animal welfare, and providing greater animal comfort [1,2]. Among them, the compost-bedded pack barn (CBPB) housing system, which is characterized by an open bedding area, without individual stalls, and with a bedding surface covered with organic material, is one of the most used confinement systems for dairy cows. The bedding area of the CBPB is used as a resting area for dairy cows and is in constant composting activity [3,4].

The composting process occurs through the degradation of animal wastes and bedding materials, which serves as a source of protein (nitrogen) and energy (carbon) for bacteria present in the composting material [5]. When this process occurs efficiently, the ideal temperatures for bedding composting are reached (>40 °C), which optimizes the process of water evaporation to control the bedding moisture content (40 to 60%) [6,7]. On the other hand, controlling bedding moisture is one of the most challenging aspects in CBPB systems [8,9] because it is influenced by several factors, such as environmental temperature, ventilation speed, moisture content of the incoming air, and stocking density [3,10].

A recent study reported that bedding moisture content and microbiological characteristics of a CBPB were positively associated with the mastitis occurrence in dairy herds [9]. When bedding moisture is not controlled, the cow’s cleanliness is compromised by the attachment and adherence of the dirt materials to the teat skin and udder, resulting in an increase in the risk of mastitis [8]. The exposure of teats to dirty bedding materials favors the growth and contamination by environmental pathogens, which can lead to the contamination of raw milk and changes in milk composition and quality [11,12].

The most significant challenge in a CBPB is to manage the bedding in extreme conditions, such as during rainy and humid winters, where microbial activity is lower, due to microclimatic conditions, affecting the quality of the bedding [13] and consequently impacting animal health and milk quality. The research problem was to provide information on how bedding characteristics can affect cow udder health and milk quality.

Monitoring the CBPB system in field studies will provide a deeper understanding of (a) the association between CBPB characteristics and milk quality and composition and (b) the association between subclinical mastitis (SCM) occurrence and physical–chemical and microbiological characteristics of bedding material. As a practical objective, our study proposes to provide results that can be used to guide decision-making within farms that use CBPBs, especially regarding bedding management strategies.

## 2. Materials and Methods

### 2.1. Herd Selection and Study Protocols

A prospective longitudinal study was conducted in dairy herds in Castro and Piraí do Sul, Paraná State, Brazil. A total of eight herds were selected via convenience sampling and the availability and interest level of the dairy farmer to participate in the study. Specific criteria for herd inclusion were lactating cows housed in the CBPB system and the number of lactating cows (>50 cows). Over a six-month period (September 2021 to February 2022), these dairy herds were visited on a monthly basis for bedding sampling, data collection on bulk tank milk quality, and cow udder health. 

The farms visited had piped milking, with a herringbone milking parlor, except farm G, which had a parallel system. The breeds used on the farms were Holstein, Jersey, and crossbred (Holstein × Jersey). 

The average number of lactating cows per farm was 142 (ranging from 70 to 237; Table 1), average milk production was 31 ± 5.3, days in milk was 177 ± 21, and parity was 2.4 ± 0.67. The cows remained housed in the CBPB system during the entire studied period and were fed according to the nutritional management of each herd.

The dairy herds were not evaluated before the start of the study for pre-existing problems such as lameness or any other illness or condition that could harm their animal welfare. Likewise, during data collection, these types of occurrences were not recorded. Animal welfare was evaluated by accessing thermal stress, inferred through temperature–humidity index data.

The stocking density (m²/cow) was calculated by dividing the total square meters of bedding by the number of cows housed. Additional information related to bedding management (e.g., bedding type, tilling frequency, and implement used) were recorded. De-tailed characteristics from the selected dairy herds are shown in Table 1. Additional details on farm structures are shown in Table S1 in the Supplementary Material.

### 2.2. Fans Wind Speed and Meteorological Conditions Data

Wind speed from the fans was measured using a thermal anemometer (AK800A, AKSO, São Leopoldo, Brazil). The thermo-anemometer was positioned below the low-velocity and high-volume (HVLS) air fans and inclined toward the high-velocity and low-volume (LVHS) air fans to simulate the wind speed experienced by the cows and reaching the bedding area.

Temperature (T°) and relative humidity (RH) inside each CBPB housing system were recorded using a datalogger with a USB connection (AK172, AKSO, São Leopoldo, Brazil). The datalogger was placed in the center of each CBPB system (about 1.8 m high), staying close to the cows but out of their reach. Temperature and RH data were recorded every 30 min. At each visit, data for the corresponding month were collected, and the datalogger was reprogrammed to monitor the following month. These data were collected with the aim of verifying microclimatic conditions and calculating the temperature–humidity index (THI), using the formula of Mader et al. [14], an indicator for classifying the thermal comfort of dairy cows.

The data collection period coincides with the spring (September to December) and part of the summer (December to March) seasons. Summers and spring are characterized by high temperatures, reaching up to 26 °C on average. However, spring days have presented more intense but less frequent minimum temperatures, while maximum temperatures have been more intense and frequent, with increases between 0.3 °C and 0.6 °C/decade in spring and between 0.1 °C and 0.4 °C/decade on the annual scale being recorded [15]. 

Based on our records, we observed an average T° and RH of 19.65 °C and 79.91%, respectively (Table S2).

### 2.3. Bedding Sampling and Milk Data Collection

Monthly, the bedding area was assessed using a grid pattern consisting of nine squares according to the methodology described by Albino et al. [16]. In the center of each square, the bedding temperature was measured at various depths layers (surface, 10, 20, and 30 cm deep). The surface temperature was measured using an 8:1 infrared thermometer (Simpla T155, AKSO, São Leopoldo, Brazil), while the temperatures at 10, 20, and 30 cm depth were measured using a skewer-type digital thermometer (HI145-00, Hanna Instruments, Tamboré Barueri, Brazil). Bedding samples (approximately 1 kg) were collected from each square and, at the end, were combined to create a representative sample (~300 g) of the entire bedding area. These bedding samples were packed in sterile plastic bags and kept frozen (−20 °C) for future analysis.

Results of bulk tank milk composition (fat, protein, total solids, milk urea nitrogen (MUN), somatic cell count (SCC), and standard plate count (SPC)) were provided by the farmers, according to monthly herd records. For monthly analyses of the quality and composition of milk from the tank, samples were taken by the company responsible for collecting milk on the farm and carried out by PARLPR—Programa de Análise de Rebanhos Leiteiros do Paraná (Dairy Herd Analysis Program from Paraná State)—of the APCBRH—Associação Paranaense Criadores de Bovinos da Raça Holandesa. Sampling for SCC analysis was carried out by employees from each farm and sent to PARLPR. PARLPR is a member of the Brazilian Network of Milk Quality Control Laboratories—RBQL—and is accredited by the Ministry of Agriculture, Livestock and Supply—MAPA. The analyses were carried out in accordance with official standards. The analyses were carried out using Fourier Transform Infrared Spectroscopy (FTIR) for milk fat, protein, total solids, and urea nitrogen and flow cytometry methods for the SCC and SPC. Milk fat, protein, and total solids were determined using Bentley model 2000 (Bentley Instruments Inc., Chaska, MN, USA) equipment, and urea nitrogen was determined with Bentley Instruments^®^ ChemSpec150 equipment. The milk SCC and SPC were obtained using Somacount 500 equipment (Bentley Instruments Inc., Chaska, MN, USA) and Bactocount^®^ IBC equipment (Bentley Instruments, Chaska, MN, USA), respectively. The SPC quantifies the total number of aerobic bacteria in raw milk.

### 2.4. Bedding Analysis

Bedding samples were subjected to the analyses of dry matter (DM; %), pH, carbon-to-nitrogen (C/N) ratio, and microbiological counting. The DM analyses were performed according to the methodology described by the AOAC (1990) [17], where bedding samples were weighed (initial weigh) and placed in a forced air circulation oven at 65 °C for 72 h. After that, samples were weighed again (final weigh). DM content was obtained by the difference between initial and final weight, while the moisture content of each sample was obtained using the following formula: $Moisture\left(\%\right)=100-\%DM$.

For analyses of pH and C/N, the samples were ground using Willey Will type (Standard Model N0.3, Philadelphia, PA, USA) equipment, with a 1 mm mesh sieve. The pH measurement was performed according to Marques [18] with some modifications. Specifically, 5 g of ground sample was mixed with 50 mL of distilled water and shaken for 15 min. Afterwards, the sample was allowed to rest for 30 min, followed by the pH measurement using a pH meter (Q400AS, Quimis, Diadema, Brazil). To determine C/N, the sample was first passed through a 0.25 mm sieve. After 12 mg of each sample was weighed on a precision scale (Sartorius brand) on a tin plate (Sn), the sample was wrapped, forming a kind of capsule, after being pressed. The determination of C/N was carried out simultaneously through dry combustion, at a temperature of 975 °C using Vario EL III (Elementar Analys en system, Hanau, Germany) equipment according to Dieckow et al. [19].

Bedding microbiological analyses were performed according to Zdanowicz et al. [20]. Briefly, 10 g of a bedding sample was diluted in 90 mL of peptone water (0.1%), followed by serial dilutions. Then, 100 mL of the dilution (10^−1^ to 10^−6^) was streaked onto blood agar (Oxoid, Basingstoke, UK), MacConkey’s medium (KASVI, São José dos Pinhais, Brazil), and Edward’s modified medium (Oxoid, Basingstoke, UK) for total aerobic bacteria, Gram-negative bacteria, and streptococci and streptococci-like organism (SSLO) counts, respectively. For presumptive staphylococci counting, the Vogel–Johnson culture medium (Acumedia, Lansing, MI, USA) was used [21]. For the MacConkey plates, lactose-fermenting (pink) colonies (e.g., presumptive coliforms) and all other colonies (e.g., non-coliforms) were counted together and reported as Gram-negative bacteria [22]. For the Edward’s plates, blue/black colonies were counted together and reported as SSLOs. Finally, for the Vogel–Johnson plates, translucent to black colonies surrounded or not by yellow zones were counted, and results were reported as presumptive staphylococci. No additional tests were performed for pathogen confirmation. All culture media were prepared according to the manufacturers’ instructions. For all microbiological tests, a 100 µL inoculum was placed in the center of each plate and spread evenly over the entire surface of the plate. Afterwards, plates were aerobically incubated at 37 °C for 24 h. Bedding bacteria counting was performed manually, and the results are expressed as log10 cfu/g.

### 2.5. Data Analysis

The data were entered into Excel spreadsheets (Microsoft Office, 2016) and checked for consistency before the data analysis. Initially, an exploratory analysis of the bedding variables (moisture, temperature, pH, and C/N ratio), environment (T° and RH), and milk quality and composition (SCC, SPC, fat, protein, and MUN) was performed. The results of the Pearson correlation coefficients showed high correlation (r > 0.93) between temperatures at 10, 20, and 30 cm deep. Therefore, only the surface temperature and the temperature at 30 cm deep were used for further analyses. Regarding milk composition, total solids showed a high correlation (r = 0.96) with fat and protein and was removed from further analysis. This first analysis step was performed to address the issue of multicollinearity in a subsequent canonical correlation analysis (CCA).

The second stage of the analysis used the CCA to identify the relationship between the groups of variables studied. CCA assessed the correlation between a linear combination of a set of response variables (*X_n_*) and a linear combination of another set of predictive variables (*W_n_*) [23,24], and those linear combinations can be defined as follows:

$$U_{i}=a_{i1}X_{1}+a_{i2}X_{2}\ldots a_{in}X_{n}$$

$$V_{i}=b_{i1}w_{1}+b_{i2}w_{2}\ldots b_{im}w_{m}$$

where $U_{i}$ and $V_{i}$ are the first pair of canonical variables associated with the first canonical correlation; $a_{in}$ and $b_{im}$ are canonical coefficients; and $X_{n}$ and $w_{m}$ are the original variables in the groups. Data interpretation was based on the Pearson correlation coefficients between the original variables and their canonical variables.

For the CCA analysis, groups of variables were formed from their original variables as follows: milk composition group (SCC, SPC, fat, protein, and MUN), bedding characteristic group (moisture, surface bedding temperature (ST), temperature at 30 cm depth (T30), pH, carbon (C), nitrogen (N), and C/N), microbiology counting group (total aerobic bacteria, Gram-negative bacteria, SSLOs, and presumptive staphylococci) and environment group (T° environment and RH). The CCA analysis was performed between these groups: milk group × bedding (41 observations); milk group × microbiology group (47 observations); milk group × environment group (38 observations); bedding group × microbiology group (50 observations), bedding group × environment group (39 observations), and microbiology group × environment group (46 observations).

To determine and classify the canonical correlation degrees, we followed the recommended intervals proposed by De Menezes et al. [25]: <0.05: very weak; 0.05 to 0.20: weak; from 0.20 to 0.30: weak to moderate; from 0.30 to 0.60: moderate; from 0.60 to 0.80: moderate to strong; from 0.80 to 0.95: strong; above 0.95: very strong.

SCM cases were considered when cows presented ≥200,000 cells/mL [26]. The prevalence of SCM (%) was defined as the number of cows with an SCC ≥ 200,000 cells/mL divided by the total number of tested cows on a given test day [27]. The incidence of SCM was defined as the number of cows whose SCC increased from <200,000 cells/mL to ≥200,000 cells/mL on two consecutive test days divided by the number of cows whose SCC was <200,000 cells/mL on the previous test day [8].

Multiple linear regression models with repeated measures were used to identify associations between bedding characteristics and the prevalence and incidence of SCM. The explanatory variables of mastitis prevalence and incidence included moisture content, C/N, pH, ST, T30, and microbiological count (total aerobic bacteria, Gram-negative bacteria, SSLOs, presumptive staphylococci). Initially, variables were tested one-by-one, and only variables with *p* < 0.20 were included and evaluated for model selection. Then, only variables with *p* ≤ 0.10 were kept in the final model. The farms were considered as the experimental unit. Assumption of residual normality was tested for both the SCM prevalence and incidence models. For SCM incidence, a lognormal distribution was used because residuals had no normal distribution. 

Bedding T30 was the only variable significant in the final model of the SCM prevalence, while the surface bedding temperature was the only variable significant in the SCM incidence model. So, both variables were also tested for polynomial effects. The surface bedding temperature had a second-order polynomial effect on SCM incidence. For this, a point of maximum response was calculated as −b/2 × c, where b is the first order coefficient term, and c is the second order coefficient term of the regression.

Canonical correlation analysis was performed using the SAS PROC CANCORR, while regression analysis was performed using SAS PROC GLIMMIX (SAS Institute, Cary, NC, USA) [28]. Statistical significance was declared when *p* ≤ 0.06.

## 3. Results

### 3.1. Milk, Bedding, and Environmental Characteristics

The medians of the SCC and SPC were 233 × 10^3^ cells/mL and 7 × 10^3^ cfu/mL, respectively (Table 2). The average fat content of milk was 3.97%, while the protein content was 3.36%. The mean total solids content was 12.92%, and the MUN was 13.57 mg/dL.

Concerning bedding variables, the moisture content was 56.42%. The surface temperatures at 10, 20, and 30 cm depths were 20.91 °C, 30.35 °C, 33.45 °C, and 35.08 °C, respectively. The mean pH was 9.87, and the C/N ratio was 15.41. The bedding microbiological count was >6.0 log^10^ cfu/g for all microbial groups evaluated (Table 2).

The microclimate conditions showed a thermal amplitude of 6 °C, with an average temperature of 19.71 °C. The RH amplitude was higher, around 19.76%, ranging from 69.54 to 89.30%, with an average of 80.04% (Table 2).

**Figure 1:**
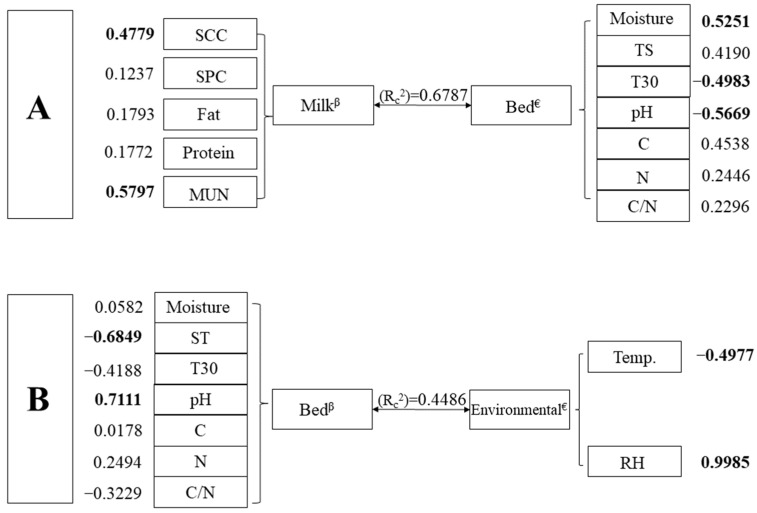
Graphical representation of the first canonical correlation function of each pair of canonical variables that had a relationship between the studied indicators and the Pearson correlation coefficients between the original variables and their canonical variables. Numbers in bold represent the variables with the highest canonical coefficients. Note: The letter A represents the correlations obtained between the milk and bedding groups. The letter B represents the correlations obtained between the bedding and environment group. SCC: somatic cell count; SPC: standard plate count; ST: surface temperature; T30: temperature at 30 cm depth; C/N: carbon/nitrogen ratio; Temp.: air temperature; RH: relative humidity. ^€^ Independent variable; ^β^ dependent variable.

Two of the six canonical correlations evaluated between the studied indicators were significant (Table 3). The results of the CCA between the milk group (dependent variables) and bedding group (independent variables) were significant (*p* = 0.0004) and showed a r_c_ = 0.82 and an $R_{2}^{C}$ = 0.68 (Figure 1). Specifically, moisture (r = 0.52), pH (r = −0.57), and temperature at 30 cm depth (r = 0.50) of the bedding were the variables with the highest values of correlation coefficients with their canonical variables influencing the milk’s SCC (r = 0.48) and MUN (r = 0.58). The linear combination of bedding variables explained about 62.71% of the variation in milk composition and quality. In addition, the CCA between the bedding group (dependent variables) and the environmental group (independent variables) was significant (*p* = 0.0222) and presented r_c_ = 0.67 and $R_{2}^{C}$ = 0.45. The microclimate variables air temperature (r = −0.50) and relative humidity (r = 0.99) obtained moderate and high canonical load, respectively, influencing the bedding temperature and pH variables. This canonical correlation showed that the influence of environmental variables could explain 77.5% of the variation in the bedding parameters (Figure 1). 

### 3.2. Subclinical Mastitis Prevalence and Incidence

**Figure 2:**
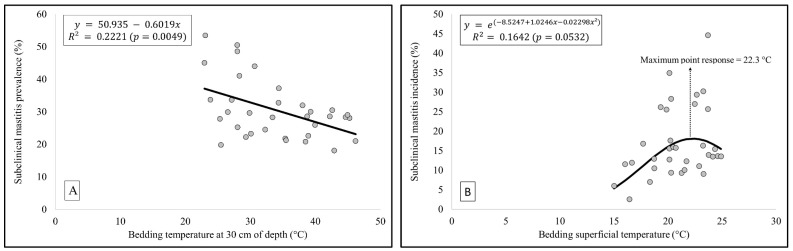
Prediction models of prevalence (**A**) and incidence (**B**) of subclinical mastitis from eight dairy herds housed in a CBPB system. Points represent the monthly prevalence and incidence values of subclinical mastitis on each farm. Lines indicate the models’ prediction values.

The median prevalence of SCM was 28.6%, while incidence was 13.8%. Only bed-ding temperature at a 30 cm depth was significant on the model for predicting prevalence of SCM (*p* = 0.0049; R^2^ = 0.22; Figure 2A). There was a linear relationship between temperature at 30 cm depth and the SCM prevalence, where an increase of 1 °C in the temperature at 30 cm depth resulted in a reduction of 0.6% in prevalence of SCM (Figure 2A). This model explained 22% of the total data variation regarding the prevalence of SCM (Figure 2A). Regarding the incidence of SCM, there was an effect of the bedding surface temperature, although it was marginally significant (*p* = 0.0532; R^2^ = 0.16; Figure 2B), following a second order polynomial one indicating a curvilinear relationship. Because the SCM incidence model has a lognormal distribution, a retransformation using an exponential function was needed after solving the formula (Figure 2B). The surface bedding temperature increased the incidence of SCM until 22.3 °C, reaching a response peak of ~18.1% of maximum incidence and decreasing beyond that point (Figure 2B). This model explained 16% of the total variation regarding the incidence of SCM (Figure 2B). 

## 4. Discussion

In recent years, research related to the CBPB system has been growing worldwide due to its positive impacts on cow health, well-being, longevity, and productivity. Nevertheless, inadequate bedding management in the CBPB system can negatively affect cows’ udder health and milk quality. Remarkably, few studies have evaluated the association between milk quality and the various factors that affect bedding characteristics. The aim of this study was to identify factors associated with milk quality and mastitis occurrence in dairy herds housed in a CBPB.

Our results showed that approximately 62.71% of the variation in milk composition and quality could be explained by the bedding variables, while approximately 77.50% of the variation in bedding parameters could be explained by environmental variation. These results corroborate the findings of Leso et al. [29], which reported that these two factors, in addition to wind speed, could explain about 70% of the variance in the bedding drying rates.

Based on our results, there was a direct relationship between the bedding pH and RH, but the same did not occur with the air temperature, as pH tended to be reduced with higher ambient temperature. The relationship between these variables could be an indirect effect because RH influences the moisture content of the bedding. In our study, it was observed that wet bedding was more compacted, contributing to a greater accumulation of ammonia in the barn and an increase in pH. Bewley et al. [30] reported that when the smell of ammonia was noticeable, the C/N was below 25:1, hindering the process of microbial activity and heat production in the bedding. Additionally, our results are in line with Shogor et al. [31], who described that bedding used for a long period tended to become alkaline, with an average pH of 9.36. Another factor that may have directly influenced the pH is the bedding temperature. Zhang et al. [32] found a high negative correlation (r = −0.956) between the bedding temperature and pH during the summer, and Oliveira et al. [33] reported that the pH had a uniform distribution along the bedding profile from the decomposition of organic matter. However, bedding presents a stratification of profiles [34], with uniformity found in layers that present the same microbial activity condition, whereas differences in temperature and pH of the bedding may exist among different layers. In our study, we observed that the temperature of the bedding presents a uniform distribution across layers because a high correlation was found between the temperatures verified at 10, 20, and 30 cm depth (r > 0.93). However, no evidence was found in the existing literature that RH and air temperature directly influence the pH of the bedding, which highlights the need for further studies to elucidate this relationship.

The results of the CCA indicated that pH, T30, and moisture were the primary variables affecting some milk characteristics, mainly the SCC and MUN. The relationship between the humidity and bedding temperature is inverse, with high bedding temperatures generating an active composting process and promoting drying of the bedding [29]. In addition, the bacterial counts, especially those causing mastitis [35], can be reduced when bedding temperatures are maintained between 54 and 65 °C [36]. However, to sustain microbial activity, maintaining an adequate humidity in the bedding (40–60%) is essential [37]. The effects of bedding moisture on the mammary gland health of dairy cows have been previously reported [8,20,33,36,38]. Excessively wet bedding, which is primarily associated with excess of manure and urine, can increase the risk of udder and teat contamination, resulting in high incidence of mastitis caused by environmental pathogens [36,38,39]. Therefore, it is recommended to maintain low humidity (<50%) or dry the bedding surface, aiming to provide adequate hygienic conditions for the cows and to reduce the incidence of clinical mastitis [9,33]. 

The median bulk tank SCC observed in this study (233 × 10^3^ cells/mL) is lower than those reported by other studies involving a CBPB in the southern region of Brazil, which described an average SCC > 340 × 10^3^ cells/mL [40,41,42]. These results suggests that the risk of mastitis and increased SCC in bulk tank milk in CBPB systems could be reduced [6,35] when adequate management is carried out, including thorough turning of the bedding, adequate animal stocking density, appropriate environmental conditions (temperature, RH, and wind speed). Bedding characteristics, such as animal density, bedding humidity, and animal cleanliness, can influence the risk of mastitis and impact milk quality [8]. Nogara et al. [42] found a moderate to weak correlation between bedding moisture and dirt score (r = 0.39), space per cow (r = −0.37), and pH (r = 0.33). Among these factors, bedding moisture was considered the primary predictor associated with the incidence of clinical mastitis [8]. These results corroborate the findings of Nogara et al. [42], where bedding moisture and pH were the most critical variables in explaining variations in milk composition and quality. Bewley et al. [30] reported that even with an excellent composting process, many environmental pathogens (>9.1 million cells/cm^3^) that cause mastitis can be found on the bedding surface. 

Among the evaluated milk composition variables, only the MUN was affected by bedding characteristics. In the present study, the average MUN value was 13.57 mg/dL, which is similar (14.45 mg/dL) to the value reported in dairy herds from Paraná state, Brazil [43]. It is worth mentioning that the suggested range for this parameter varies between 10 and 14 mg/dL [44], with optimal levels around 11.7 mg/dL [45]. In addition to diet-related factors, variation in MUN content may occur due to abnormal milk composition, freshly calved cows, and milk with a high SCC [46], which may be the most likely explanation for the effect found in our study. Also, days in milk, parity, genetics, body weight of cows, and the procedures used for milk sample collection could also contribute to the variations in MUN content [47,48].

The breed of cows may also have contributed to the observed high levels of MUN in our study since high urea levels came from two farms (D and H) that used exclusively Jersey cows. Farm D in all evaluation months presented a MUN greater than 18 mg/dL. The effect of different breed groups (Holstein, Jersey, Brown Swiss, and crossbred cattle) on MUN values was previously described, with Brown Swiss cows presenting the highest values (17.62 mg/dL), followed by the Jersey cows (16.12 mg/dL), while the Holstein cows had the lowest content (14.18 mg/dL) [43]. This variation in MUN values can be explained by the fact that the Holstein breed has a greater milk yield, which leads to a higher efficiency in using amino acids and consequently lower levels of MUN in the milk compared to the other breeds.

Additionally, in these two specific farms (D and H), the highest SCC values were found (mean 396 and 525 × 10^3^ cells/mL, respectively). In these farms, the bedding humidity was 53.65 and 65.42%, respectively. In farm D, there was no artificial ventilation system, the barn height was 5.06 m, with a stocking density of 8.47 m²/cow. Cases of thermal stress, verified by THI, were more frequent on this farm during the experimental period, as the farm had higher maximum THI averages (Supplementary Material). From these data, it can be inferred that these herds experienced heat stress events, mainly from December to February 2022. As an example, farm D experienced an air temperature of 21.36 °C and an RH of 80.44%, resulting in a THI of 69.11, which indicates a mild stress condition, according to Rodriguez-Venegas et al. [49]. However, for Collier et al. [50], production losses in dairy cattle were already verified with a THI of 68. The level of heat stress experienced by cows is a combination of the effects of air temperature and humidity, which are calculated using the temperature–humidity index (THI) [51].

Doska et al. [43] reported that environmental factors could reduce the efficiency of dietary nitrogen utilization. This finding may provide a possible explanation for the elevated levels of MUN observed in our study (20.10 mg/dL—maximum value). However, similar results were not found in the literature, which suggests the need for new studies investigating the parameters of the CBPB bedding, thermal stress, and SCC with MUN concentrations.

The model results for predicting the SCM prevalence showed that a 1 °C increase in T30 was associated with a 0.6% reduction in the SCM prevalence, explaining 22% of the data variation. Considering that mastitis is a complex and multifactorial disease [52], the result that >20% of the variation in SCM prevalence data could be associated with the CBPB bedding reinforces the importance of keeping the bedding active and dry and maintaining high temperatures to inactivate pathogens that are harmful to the mammary gland health [35,36]. To reduce the overall microbial load, such as streptococci and total coliforms in the bedding, and to reduce the exposure of the udder to these pathogens, it is essential that the bedding temperature remains between 55 and 60 °C [12,38].

A positive association between bedding variables (e.g., microbiological counts) and the SCM prevalence in herds housed in a CBPB has been reported [9]. Despite this, previous studies did not find any CBPB bedding variables associated with the incidence or prevalence of this type of mastitis in the studied herds [8]. However, both authors report high microbiological counts in the bedding, which agrees with our bedding microbiological results. It is important to mention that the culture media used in this study and in previous research [8,9] were not designed for the analysis of bedding samples. Therefore, this limitation could lead to misclassification of the studied bacterial groups (e.g., streptococci and staphylococci) considering no confirmation step was performed. The use of a specific selective medium for pathogen enumeration of bedding materials should be considered in future research, given the association of bedding bacterial counts and mastitis in dairy cows [9]. In addition, establishing a standard procedure for microbiological analysis of bedding can aid in comparisons between studies. These methodologies should include not only the culture media to be used but also the recommended incubation temperatures for the pathogens of interest.

The bedding surface temperature significantly affected the incidence of SCM, given the positive association between the variables. When the surface temperature increased to 22.3 °C, the incidence followed the same pattern, reaching a peak of 18.1%. The model used in this study explained 16% of the total variation in the data regarding the incidence of SCM, which can be considered a very substantial proportion, considering mastitis is a multifactorial disease [53,54]. This significant association can be explained by the contamination of the bedding surface layer. Bewley et al. [30] point out that many environmental pathogens that cause mastitis can be found on the bedding surface, increasing the risk of udder and teat exposure and consequently the risk of mastitis [8,36,39]. Therefore, it is essential to provide a dry surface for cows to rest with the aim of preventing mastitis in dairy cows [33]. However, further studies are needed to determine why the incidence peak of SCM occurred at 22.3 °C. Our hypothesis is that the temperature range of 15 to 22.3 °C is favorable for the growth of opportunistic bacteria. For example, *S. uberis* has an optimal multiplication temperature above 21 °C and is frequently isolated in raw milk samples [12,55,56,57] because of the risk of fecal contamination of teats and udder skin. 

One limitation of this study is that we analyzed the occurrence of mastitis without previously accessing the profile of causative pathogens. Previous studies reported that the presence of contagious pathogens in the evaluated herds could lead to an experimental bias [8,9]. Therefore, we emphasize the importance of determining the profile of mastitis-causing pathogens as a criterion to select CBPB herds in future research. Despite this limitation, our results can provide valuable insights for guiding management strategies in CBPB dairy herds.

## 5. Conclusions

This study demonstrated that bedding characteristics significantly affect the quality and composition of milk from dairy farms using the CBPB system. Approximately 62.71% of the variation in milk composition and quality could be explained by bedding characteristics, with moisture, T30, and bedding pH as the most critical factors. The bedding temperature is one of the most influential variables for assessing the quality of composting and, consequently, the risk of SCM. A 1 °C increase in T30 resulted in a 0.6% reduction in the SCM prevalence. In contrast, the relationship between the bedding surface temperature and SCM incidence displayed a curvilinear relationship, reaching a peak of 18.1% of SCM at 22.3 °C. However, through our data we can see that the milk SCC was still higher in farms that had high-humidity bedding (65.42%). These data reinforce the importance of working seriously on bedding management, aiming for a faster bedding drying rate so that humidity can be controlled within the ideal range of 40 to 60%, and reaching adequate temperatures (>45 °C) for the process of composting. Our results can support decision makers in the dairy industry who seek strategies for bedding management, milk quality, and mastitis control.

## Figures and Tables

**Table 1:** Characteristics of the CBPB from eight dairy herds evaluated over six months.

Farm	Lactating Cows (*n*)	Breed	Stocking Density (m^2^/cow)	Bedding Type	Tilling Frequency (Times/Day)	Tillage Equipment	Fan Type
A	200 ± 17.09	Holstein	9.04	Sawdust and shavings	2	Rotating hoe	HVLS ^1^
B	123.80 ± 3.56	Holstein	11.26	Sawdust and shavings	2	Rotating hoe and Cultivator	LVHS ^2^
C	237 ± 15.29	Holstein	16.13	Sawdust and shavings	2	Rotating hoe	LVHS
D	125.86 ± 4.49	Jersey	8.47	Sawdust and shavings	2	Cultivator	-
E	69.80 ± 8.58	Holstein	12.60	Sawdust and shavings	2	Cultivator	LVHS
F	174.57 ± 10.47	Holstein	13.10	Sawdust and shavings	2	Rotating hoe and Cultivator	HVLS
G	92.75 ± 7.80	Holstein	14.40	Sawdust and shavings	2	Rotating hoe	LVHS
H	108 ± 9.84	Jersey	13.81	Sawdust and shavings	2	Rotating hoe	LVHS
Mean	141.67 ± 57.35	-	12.35	-	2	-	-

^1^ Low-velocity and high-volume air fans; ^2^ high-velocity and low-volume air fans.

**Table 2:** Descriptive statistics of milk quality and composition, bedding, and environmental variables from eight dairy herds housed in a CBPB.

Variable	Category ^1^	N ^2^	Minimum	Mean	Median	Maximum
SCC ^3^ (×10^3^ cells/mL)	**Milk**	48	129.67	281.79	232.67	599.67
SPC ^4^ (×10^3^ cfu/mL)		48	3.40	10.54	7.23	48.29
Fat (%)		48	3.34	3.97	3.86	4.74
Protein (%)		48	3.03	3.36	3.30	3.75
Total solids (%)		48	12.12	12.91	12.79	14.04
MUN ^5^ (mg/dL)		48	8.63	13.57	12.59	20.10
Moisture (%)	**Bedding**	58	29.91	56.42	59.45	71.92
ST ^6^ (°C)		57	15.00	20.91	20.55	25.02
T10 ^7^ (°C)		59	18.10	30.35	29.30	46.92
T20 ^8^ (°C)		59	19.43	33.45	31.03	54.77
T30 ^9^ (°C)		58	21.98	35.08	32.84	58.86
pH		53	9.25	9.87	10.01	10.58
C ^10^ (%)		53	18.24	29.31	30.37	35.52
N ^11^ (%)		53	1.05	1.95	1.90	3.95
C/N ^12^		53	6.92	15.41	15.71	19.40
Stocking density (m²/cow)		14	5.14	12.50	13.46	16.99
Presumptive staphylococci (log_10_ cfu/mL)	**Bedding microbiology counting**	58	4.60	6.33	6.28	9.38
SSLOs ^13^ (log_10_ cfu/mL)		58	4.95	6.33	6.30	8.84
Gram-negative bacteria (log_10_ cfu/mL)		58	1.49	5.09	5.20	7.15
Total aerobic bacteria (log_10_ cfu/mL)		58	6.00	7.58	7.53	9.86
Temperature (°C)	**Microclimate**	48	16.32	19.71	20.46	22.30
Relative humidity (%)		48	69.54	80.04	80.44	89.30
Wind speed (m/s)		29	1.10	3.25	2.60	6.50

^1^ Group of variables; ^2^ number of observations; ^3^ SCC: somatic cell count; ^4^ SPC: standard plate count; ^5^ MUN: milk urea nitrogen; ^6^ ST: surface temperature; ^7^ T10: temperature at 10 cm depth; ^8^ T20: temperature at 20 cm depth; ^9^ T30 temperature at 30 cm depth; ^10^ C: carbon; ^11^ N: nitrogen; ^12^ C/N: carbon/nitrogen ratio; ^13^ streptococci and streptococci-like organisms.

**Table 3:** Canonical correlations between environmental, bedding and milk quality, and composition variables.

Canonical Correlation	(r_c_) ^1^	$\left(R_{2}^{C}\right)$ ^2^	Proportion	*p*-Value
Milk ^β 3^ × Bedding ^€ 4^	0.8236	0.6787	0.6271	0.0004
Milk ^β^ × Microbiology ^€ 5^	0.5131	0.2633	0.5459	0.2606
Milk ^β^ × Microclimate ^€ 6^	0.5487	0.3011	0.7691	0.1055
Bedding ^β^ × Microbiology ^€^	0.5377	0.2887	0.5496	0.4779
Bedding ^β^ × Microclimate ^€^	0.6697	0.4486	0.7750	0.0222
Microbiology ^β^ × Microclimate ^€^	0.3249	0.1056	0.8159	0.6788

^€^ Independent variable; ^β^ dependent variable; ^1^ correlation coefficient; ^2^ canonical correlation; ^3^ somatic cell count, standard plate count, fat, protein, milk urea nitrogen; ^4^ moisture, surface temperature, temperature at 30 cm depth, pH, carbon, nitrogen, carbon/nitrogen ratio; ^5^ total aerobic bacteria, Gram-negative bacteria, SSLOs, presumptive staphylococci; ^6^ air temperature and relative humidity.

## Data Availability

The dataset can be requested from the corresponding author.

## Supplementary Materials

The following supporting information can be downloaded at: https://www.mdpi.com/article/10.3390/ani13233638/s1, Table S1: Description of the structural characteristics of the CBPB from eight dairy herds evaluated over six months.; Table S2: Microclimatic conditions and calculating the temperature–humidity index (THI) to infer heat stress in dairy cows from eight herds.
