# Supplementary material for: Factors Influencing Milk Quality and Subclinical Mastitis in Dairy Herds Housed in Compost-Bedded Pack Barn System
Source: Animals (Basel). 2023 Nov 24;13(23):3638. doi: 10.3390/ani13233638 (PMC10705328; doi:10.3390/ani13233638)
Supplement: Supplementary file 1 [file animals-13-03638-s001.zip › animals-2674320-supplementary.pdf]

Table S1. Description of the structural characteristics of the CBPB from eight dairy herds evaluated over six months.

Farm	Project	Curtains	Headroom height (m)	Bedding area (m^2)	Trough area (m)	Type trough area	Division into batches	Presence clerestory
A	Designed	No	5.60	1806.18	74.79	Parallel to the bed	No	Yes
B	Designed	No	5.55	1394.58	79.28	Parallel to the bed	Yes	Yes
C	Designed	No	4.98	3822.80	47.77	CBPB separate feeding	Yes	Yes
D	Adapted	No	5.06	1066.58	52.00	CBPB separate feeding	No	No
E	Designed	No	9.63	879.43	40.06	Parallel to the bed	No	Yes
F	Designed	No	8.64	2287.79	66.46	Parallel to the bed	Yes	Yes
G	Designed	No	8.74	1335.78	35.68	Parallel to the bed	No	Yes
H	Designed	No	4.57	1370.42	70.00	CBPB separate feeding	No	Yes
Mean	-	-	6.60	1745.45	-	-	-	-

Table S2. Microclimatic conditions and calculating the temperature-humidity index (THI) to infer heat stress in dairy cows from eight herds.

		**Temperature**			**Relative Humidity**			**Temperature-humidity index** [1]		
**Farm**	**Period**	**Minimum**	**Medium**	**Maximum**	**Minimum**	**Medium**	**Maximum**	**Minimum**	**Medium**	**Maximum**
A	Spring	9.65	17.13	27.18	37.75	86.35	98.31	49.70	62.64	76.06
	Spring	10.27	19.50	31.43	28.12	80.02	98.07	50.86	66.28	77.72
	Spring	9.75	20.93	30.87	27.14	75.54	97.57	49.79	68.28	79.00
	Summer	11.73	21.60	33.02	39.50	79.30	95.36	53.41	69.60	82.15
**Mean**		**10.35**	**19.79**	**30.62**	**33.13**	**80.30**	**97.33**	**50.94**	**66.70**	**66.70**
B	Spring	9.46	16.65	26.54	36.47	87.62	99.32	49.34	61.86	74.54
	Spring	9.53	18.72	30.95	29.96	82.38	98.80	49.64	65.11	77.22
	Spring	10.46	20.39	30.69	27.59	76.76	97.71	51.23	67.51	78.24
	Summer	12.50	20.99	32.12	41.02	81.55	95.77	57.78	68.77	81.29
	Summer	14.69	21.62	31.23	44.87	77.65	89.28	58.56	69.51	81.26
**Mean**		**11.33**	**19.67**	**30.31**	**35.98**	**81.19**	**96.18**	**53.31**	**66.55**	**78.51**
C	Spring	9.28	17.06	27.30	39.35	83.54	96.27	49.32	62.13	75.75
	Spring	10.46	19.32	30.74	25.70	77.60	93.95	51.43	65.36	78.00
	Spring	11.58	20.60	29.71	29.81	73.32	94.21	53.25	67.06	77.81
	Summer	12.12	21.11	30.69	41.60	80.10	94.37	54.18	68.50	80.34
	Summer	14.21	21.54	30.90	45.96	80.39	93.21	57.74	69.22	80.30
**Mean**		**11.53**	**19.923**	**29.867**	**36.48**	**78.99**	**94.40**	**53.18**	**66.45**	**78.44**
D	Spring	9.01	16.61	27.35	37.44	85.90	97.30	48.65	61.37	76.01
	Spring	8.86	18.76	33.00	25.57	80.56	97.74	48.37	64.44	79.85
	Spring	10.73	20.53	31.66	31.44	74.31	97.72	51.55	66.77	80.49
	Summer	11.37	21.09	32.63	34.22	80.96	96.20	52.76	68.38	82.16
	Summer	12.65	21.63	32.40	39.38	79.93	92.96	55.02	69.17	81.91
**Mean**		**10.52**	**19.72**	**31.41**	**33.61**	**80.33**	**96.38**	**51.27**	**66.03**	**80.08**
E	Spring	9.4	16.32	25.70	45.80	83.16	97.00	49.64	61.21	72.59
	Spring	10.3	18.31	29.00	38.50	75.76	88.00	51.31	63.93	75.59
	Spring	9.6	20.15	30.00	32.50	69.54	86.30	50.09	66.31	78.14
	Summer	12.70	20.76	30.70	47.50	76.48	88.60	55.26	67.83	81.21

	Summer	14.30	21.30	30.10	56.70	80.50	90.30	57.89	68.99	80.13
**Mean**		**11.26**	**19.37**	**29.10**	**44.20**	**77.09**	**90.04**	**52.84**	**65.65**	**77.53**
F	Spring	9.50	16.74	27.40	37.60	82.19	96.30	49.80	61.58	73.22
	Spring	10.4	18.70	28.70	34.90	76.31	91.50	51.42	64.54	76.02
	Spring	10.30	20.70	30.70	29.60	69.70	89.90	51.17	67.11	77.80
	Summer	12.60	21.23	30.50	41.80	72.97	90.30	55.02	68.14	80.29
	Summer	14.10	21.40	31.10	43.00	79.40	94.40	57.55	68.80	80.30
**Mean**		**11.38**	**19.75**	**29.68**	**37.38**	**76.11**	**92.48**	**52.99**	**66.03**	**77.53**
G	Spring	9.30	16.50	26.30	39.50	89.30	100	48.92	61.30	75.30
	Spring	9.60	18.60	31.00	33.50	84.40	100	49.59	64.50	77.80
	Spring	9.20	20.20	30.40	27.30	77.80	100	48.73	66.70	78.75
	Summer	10.90	21.03	32.50	42.80	85.23	100	51.80	68.67	82.50
	Summer	13.60	21.60	31.10	48.70	85.63	100	56.61	69.61	85.20
**Mean**		**10.52**	**19.59**	**30.26**	**38.36**	**84.47**	**100.00**	**51.13**	**66.16**	**79.91**
H	Spring	8.40	16.40	26.60	41.50	84.76	97.30	47.64	61.05	75.13
	Spring	9.60	18.56	30.40	33.00	79.86	97.10	49.83	64.29	76.51
	Spring	9.80	20.07	29.80	36.90	75.22	95.70	50.11	66.37	79.14
	Summer	12.10	20.82	31.10	42.60	81.35	96.90	54.13	68.12	80.34
	Summer	14.00	21.12	30.10	42.90	82.83	98.90	57.36	68.89	79.68
**Mean**		**10.78**	**19.39**	**29.60**	**39.38**	**80.80**	**97.18**	**51.81**	**65.74**	**78.16**
**Mean period**		**10.96**	**19.65**	**30.11**	**37.32**	**79.91**	**95.50**	**52.18**	**67.67**	**77.11**

[1] Calculated from the formula proposed by Mader et al. [1]: Temperature-humidity index (THI) = 0.8 × ambient temperature + [(% relative humidity ÷ 100) × (ambient temperature − 14.4)] + 46.4. Classification of thermal stress based on THI, according to Rodriguez-Venegas et al. [2], where: < 68 (normal); 68–71 THI (light); 72–76 THI (moderate); 77–79 THI (intense), and ≥ 80 (extreme).

References:

1. Mader, T.L.; Davis, M.S.; Brown-Brandl, T. Environmental factors influencing heat stress in feedlot cattle. *J. Anim. Sci.*, **2006**, *84*, 712-719. DOI: 10.2527/2006.843712x.

2. Rodriguez-Venegas, R.; Meza-Herrera, C.A.; Robles-Trillo, P.A.; Angel-Garcia, O.; Rivas-Madero, J.S.; Rodriguez-Martínez, R. Heat Stress Characterization in a Dairy Cattle Intensive Production Cluster under Arid Land Conditions: An Annual, Seasonal, Daily, and Minute-To-Minute, Big Data Approach. *Agriculture,* **2022**, *12*, 760. DOI: 10.3390/ agriculture12060760.
